# Clinical characteristics in *Russula subnigricans* poisoning: a retrospective study of 103 cases

**DOI:** 10.3389/ftox.2026.1815129

**Published:** 2026-04-02

**Authors:** Lei Wu, Congli Yang, Yangshan Fu, Ruiling Zuo, Xingcheng Li, Junyue Hu, Shibiao Fu, Yaowu Chen, Wenfang Zhang, Fenshuang Zheng

**Affiliations:** 1 Dali University, Dali, China; 2 The Affiliated Hospital of Yunnan University, Kunming, China; 3 Baoshan People’s Hospital, Baoshan, China; 4 The People’s Hospital of Wenshan Prefecture, Wenshan, China; 5 Mile City Traditional Chinese Medicine Hospital, Mile, China; 6 Lijiang People’s Hospital, Lijiang, China

**Keywords:** blood purification, mushroom poisoning, rhabdomyolysis, Russula subnigricans, treatment

## Abstract

**Background:**

Mushroom poisoning, a form of foodborne intoxication, poses serious life-threatening risks in severe cases. *Russula subnigricans*, a rare but highly toxic species, warrants particular attention due to its unique symptom profile and clinical manifestations.

**Methods:**

We conducted a retrospective analysis of 103 patients with confirmed highly suspected Russula subnigricans poisoning admitted to the Affiliated Hospital of Yunnan University between 2020 and 2024. Data encompassed epidemiological characteristics, clinical manifestation, laboratory findings, and treatment outcomes.

**Results:**

Patients were predominantly middle-aged (61.2%) and from Yunnan Province (93.2%), with most poisonings (93.2%) resulting from Self-picking. Symptom onset was rapid (median latency, 2.4 h), primarily featuring gastrointestinal, cardiovascular, and rhabdomyolysis-related manifestations. Laboratory findings revealed high rates of hepatotoxicity (97.1%) and myocardial injury (85.4%). A combination of conventional therapy and blood purification was employed in 60.2% of cases, yielding favorable outcomes. After treatment at Yunnan University Affiliated Hospital, the final clinical outcomes were as follows: 91 patients (88.3%) recovered and were discharged.

**Conclusion:**

*Russula subnigricans* poisoning is characterized by severe multi-organ toxicity. The adjunctive application of blood purification in combination with conventional therapy appears associated with improved clinical outcomes, although no specific antidote exists. Future research should focus on elucidating toxin mechanisms and toxicokinetics to guide targeted treatment and prevention strategies.

## 1 Background

China hosts over 1,000 species of edible mushrooms; yet it also harbors a considerable number of toxic species, with estimates reaching as high as 480, among which more than 40 are highly toxic (Xu et al., 2020). A 2024 report from the Chinese Center for Disease Control and Prevention (CDC) indicated that a total of 588 mushroom poisoning incidents occurred across 28 provinces, resulting in 13 deaths (Li et al., 2025). Mushroom poisoning is thus a major lethal foodborne disease demanding prompt emergency medicine attention.

In a multicenter assessment of mushroom poisoning conducted in Chuxiong, Yunnan Province, China, patients were categorized into three risk groups based on their vital signs: low-risk (stable vital signs, typically with an incubation period of less than 6 h), moderate-risk (symptoms including transient consciousness impairment, gastrointestinal symptoms leading to internal environment disturbance, organ dysfunction, or abnormal liver and kidney function indicators), and high-risk (unstable vital signs, impaired consciousness, organ dysfunction or failure, and severe internal environment disturbance) (Yao et al., 2023). The low-risk group involved poisoning by *Russula* species (excluding *Russula subnigricans*), whereas the high-risk group involved poisoning by *R. subnigricans*. According to the most recent classification of mushroom poisoning, six main clinical types are recognized: (1) hepatorenal injury; (2) neurotoxic; (3) myotoxic; (4) metabolic, endocrine, and related toxicity; (5) gastrointestinal irritant; and (6) miscellaneous adverse reactions (White et al., 2019). *Russula subnigricans* falls under the rapid-onset category of myotoxic mushroom poisoning.

*Russula subnigricans* poisoning has been documented worldwide. In 2015, Lin, a Japanese scholar, reported a mass poisoning event involving seven patients (Lin et al., 2015). In 2023, Chun from Japan reported a recent episode (Chun et al., 2023). In 2016, Cho from Korea reported the first case of fatal *R. subnigricans* poisoning (Cho and Han, 2016). In Hunan Province, China, 20 deaths were attributed to *Russula* subnigricans between 2014 and 2023 (Wu et al., 2025). Furthermore, data from the Chinese Center for Disease Control and Prevention (CDC) covering 2012 to 2023 identify *R. subnigricans* and *Amanita* species as the leading causes of death from mushroom poisoning (Yuan et al., 2024). Across these reports, rhabdomyolysis (including delayed-onset manifestations) was recognized as a hallmark clinical feature, with some patients also developing myocardial injury and acute kidney injury.

*Russula subnigricans* poisoning, a severe but rare form of mushroom toxicity, was investigated through a retrospective analysis of 103 suspected cases admitted to the Affiliated Hospital of Yunnan University. The clinical characteristics and onset patterns were examined, providing valuable insights for developing more effective diagnostic and treatment guidelines for suspected *R. subnigricans* poisoning cases.

## 2 Subjects and methods

### 2.1 Study subjects

We retrospectively collected clinical data from patients admitted to the Affiliated Hospital of Yunnan University for highly suspected *R. subnigricans* poisoning between January 2020 and December 2024. The inclusion and exclusion criteria were as follows:

Inclusion Criteria: 1) Highly suspected ingestion of *R. subnigricans*, as identified jointly by medical staff and patients using standard mushroom identification guides; 2) Diagnosis of mushroom poisoning meeting the criteria established in the “Clinical Expert Consensus on Diagnosis and Treatment of Mushroom Poisoning in China”; 3) Manifestation of varying degrees of poisoning symptoms in all individuals who consumed the mushrooms, and an absence of symptoms in those who did not; 4) Age ≥14 years; 5) Availability of complete clinical data.

Exclusion Criteria: 1) Concurrent ingestion of multiple species of wild mushrooms; 2) Pre-existing significant chronic diseases affecting major organs, e.g., heart, liver, or kidneys (Rationale: To improve cohort homogeneity and reduce confounding effects on clinical outcomes); 3) A known history of hepatic, cardiac, or renal dysfunction [Rationale: These conditions may alter toxin metabolism and the clinical manifestation of poisoning, thereby complicating outcome assessment]; 4) Co-exposure to any other recognized toxin or foodborne poison; 5) Incomplete clinical datasets; 6) Loss to follow-up preventing assessment of prognosis.

Note on external validity: The exclusion of individuals with significant pre-existing organ dysfunction may limit the generalizability of our findings. In real-world clinical settings, such comorbidities are common and may exacerbate both the severity and mortality risk of mushroom poisoning. Consequently, our results may underestimate the true clinical burden of *R. subnigricans* poisoning in more vulnerable patient populations.

### 2.2 Research methods

In this retrospective study, we collected data through a comprehensive review of medical records, supplemented with patient interviews. Epidemiological data included the time and location of poisoning events, the number of affected individuals, and the source of the mushrooms. Clinical data encompassed patient medical history, symptom latency period, presenting symptoms, vital signs, auxiliary examination findings, clinical management strategies, treatment procedures, and patient outcomes.

### 2.3 Definitions of clinical outcomes

The following operational definitions were used to identify key complications during hospitalization:

Rhabdomyolysis: peak creatine kinase (CK) level exceeding five times the upper limit of normal (ULN; >1550 U/L, with ULN of 310 U/L), accompanied by clinical manifestations such as muscle pain, weakness, or dark urine.

Acute kidney injury (AKI): defined according to the Kidney Disease: Improving Global Outcomes (KDIGO) 2012 criteria, i.e., an increase in serum creatinine (Cr) by ≥ 0.3 mg/dL (≥26.5 μmol/L) within 48 h, or an increase to ≥1.5 times baseline within the prior 7 days, or urine output <0.5 mL/kg/h for 6 h.

Hepatotoxicity: peak alanine aminotransferase (ALT) or aspartate aminotransferase (AST) > 3×ULN (ALT >150 U/L, AST >120 U/L, with ULN of 50 U/L and 40 U/L, respectively).

Myocardial injury: peak creatine kinase-MB (CK-MB) >2 × ULN (>50 U/L, with ULN of 25 U/L) or high-sensitivity cardiac troponin I (hs-cTnI) exceeding the 99th percentile upper reference limit.

Coagulopathy: prothrombin time (PT) or activated partial thromboplastin time (APTT) >1.5 × ULN, or international normalized ratio (INR) >1.5.

Abnormal hematology: defined as platelet count <100 × 10^9^/L, white blood cell count <3.0 × 10^9^/L or >12.0 × 10^9^/L, or hemoglobin <100 g/L.

These definitions were established prior to data analysis and applied consistently across all patients.

### 2.4 Statistical methods

All statistical analyses were run in IBM SPSS Statistics, Version 31.0. Categorical variables (e.g., sex, age, cooking method, past medical history, geographic area, symptoms, etc.) are presented as frequencies (n) and percentages (%). Continuous variables that were non-normally distributed (e.g., incubation period) are summarized as median and interquartile range (IQR; P25, P75). Inter-group comparisons of blood-purification strategies were performed with the Wilcoxon signed-rank. A two-sided p-value <0.05 was considered statistically significant. In patients receiving blood purification all 62 patients had complete data on all indicators at the two time points of “peak” and “before discharge”, and no imputation of missing values was performed.

## 3 Results

### 3.1 Epidemiological characteristics

**FIGURE 1:**
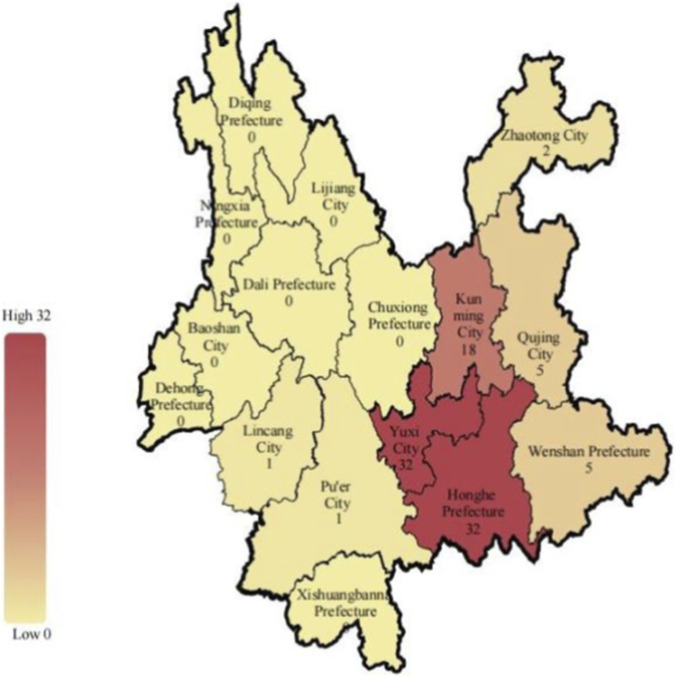
Geographic distribution of *Russula subnigricans* poisoning cases in Yunnan Province, China.

The cohort comprised 103 patients, of whom 57 (55.3%) were male. The majority of patients (86, 83.5%) had no significant comorbidities. Among the remainer, pre-existing conditions included rheumatoid arthritis (2, 1.9%), hypertension (5, 4.9%), diabetes mellitus (5, 4.9%), hepatitis B (1, 1.0%), typhoid fever (1, 1.0%), urticaria (1, 1.0%), and a history of lung tumor resection (1, 1.0%). Age distribution was as follows: 14–19 years (3, 2.9%), 20–35 years (25, 24.3%), 36–59 years (63, 61.2%), and ≥60 years (12, 11.6%). Poisoning incidents occurred predominantly in Yunnan Province (96, 93.2%) and Guizhou Province (7, 6.8%). Within Yunnan, cases were distributed across Honghe Prefecture and Yuxi City (32, 31.1%), Kunming City (18, 17.5%), Wenshan Prefecture and Qujing City (5, 4.9%), Zhaotong City (2, 1.9%), and Lincang City and Pu’er City (1, 1.0%) (Figure 1). The main source of mushrooms was self-picking (96, 93.2%), followed by market purchase (7, 6.8%). The most common preparation methods were stir-frying (67, 65.0%) and boiling (36, 35.0%). Morphological identification based on standard charts confirmed all consumed specimens ashighly suspected *R. subnigricans* (Table 1).

**TABLE 1:** Epidemiological characteristics of patients with *Russula subnigricans* poisoning.

Characteristics	n	%
Sex		
Male Female	57	55.3
	46	44.7
Age (years)		
14–19	3	2.9
20–35	25	24.3
36–59	63	61.2
≥60	12	11.6
Cooking method		
Boiling	36	35.0
Stir-frying	67	65.0
Past medical history		
Rheumatoid arthritis	2	1.9
Hypertension	5	4.9
Diabetes mellitus	5	4.9
Gout	1	1.0
Hepatitis B	1	1.0
Typhoid fever	1	1.0
History of pulmonary tumor resection	1	1.0
Urticaria	1	1.0
No significant comorbidity	86	83.5
Source of mushrooms		
Self-picking	96	93.2
Market purchase	7	6.8
Mushroom identification		
Pattern recognition	103	100.0
Species identification	0	0.0
Geographic area		
Yunnan province	96	93.2
- Honghe prefecture	32	31.1
- Yuxi city	32	31.1
- Kunming city	18	17.5
- Qujing city	5	4.9
- Wenshan prefecture	5	4.9
- Zhaotong city	2	1.9
- Lincang city	1	1.0
- Pu’er city	1	1.0
Guizhou province	7	6.8

### 3.2 Clinical manifestations

The latent period was less than 6 h in 84 patients (81.6%). Clinical manifestations varied, primarily involving the digestive, respiratory, cardiovascular, and neurological systems, along with features consistent with rhabdomyolysis. Gastrointestinal symptoms were most prevalent, including nausea and vomiting (88, 85.4%), diarrhea (21, 20.4%), and abdominal pain or bloating (19, 18.4%). Cardiovascular findings comprised chest tightness (4, 3.9%), chest pain (1, 1.0%), and palpitations (3, 3.0%). Symptoms associated with rhabdomyolysis included myalgia (54, 52.4%) and generalized fatigue (21, 20.4%). Neurological manifestations were reported as dizziness in 17 patients (16.5%), limb numbness in 4 (3.9%), and convulsions in 1 (1.0%). Respiratory manifestations, noted in 2 patients (1.9%), consisted of dyspnea.

Upon admission, poisoning severity was graded with the Poisoning Severity Score (PSS) and mushroom poisoning HOPE6 scoring system (HOPE6 score). The PSS classified 62 patients (60.2%) as severe, 11 (10.7%) as moderate, and 30 (29.1%) as mild. HOPE6 scores were distributed as 3 points in 62 (60.2%), 4 points in 18 (17.5%), 2 points in 22 (21.4%), and 1 point in 1 (1.0%) (Table 2). It should be noted that the application of these scoring systems in the context of *Russula subnigricans* poisoning remains exploratory and has not been formally validated.

**TABLE 2:** Clinical characteristics of patients with *Russula subnigricans* poisoning.

Characteristics	n	%
Latent period		
≤6 h	84	81.6
>6 h and ≤12 h	13	12.6
>12 h and ≤24 h	5	4.9
>24 h	1	1.0
Digestive system		
Nausea, vomiting	88	85.4
Abdominal pain, distention	19	18.4
Diarrhea	21	20.4
Respiratory system		
Dyspnea	2	1.9
Cardiovascular system		
Chest pain	1	1.0
Chest distress	4	3.9
Palpitation	3	3.0
Nervous system		
Limb numbness	4	3.9
Dizziness	17	16.5
Convulsion	1	1.0
Rhabdomyolysis-related symptoms		
Myalgia	54	52.4
Fatigue	21	20.4
Abnormal laboratory findings		
Hematology	88	85.4
Liver function	100	97.1
Renal function	24	23.3
Myocardial enzymes	88	85.4
Coagulation profile	13	12.6
ECG		
ST-elevation	15	14.6
ST-depression	41	39.8
Intraventricular block	5	4.9
Tachycardia	7	6.8
Other nonspecific	7	6.8
Normal	36	36.9
PSS score		
Mild	30	29.1
Moderate	11	10.7
Severe	62	60.2
Death	0	0.0
HOPE6 score		
1	1	1.0
2	22	21.3
3	62	60.2
4	18	17.5

Admission laboratory investigations revealed a high prevalence of abnormalities: deranged liver function tests (100, 97.1%), abnormal coagulation profiles (13, 12.6%), elevated myocardial enzymes (88, 85.4%), abnormal hematological parameters (88, 85.4%), and impaired renal function (24, 23.3%). Electrocardiographic (ECG) abnormalities were present in 67 patients (65.1%), including ST-segment depression (41, 39.8%), ST-segment elevation (15, 14.6%), tachycardia (7, 6.8%), intraventricular conduction blocks (5, 4.9%), and other non-specific changes (7, 6.8%). No significant ECG abnormalities were detected in the remaining 36 patients (35.0%) (Table 2).

### 3.3 Treatment and outcomes

The majority of patients (93, 90.3%) were admitted to general wards, while 6 (5.8%) required intensive care unit (ICU) admission, and 4 (3.9%) were managed in the nephrology department. A total of 41 patients (39.8%) received usual therapy, which comprised gastrointestinal decontamination (gastric lavage, activated charcoal, and/or laxatives), hepatoprotective agents, gastroprotective agents, and fluid resuscitation. The remaining 62 patients (60.2%) were managed with usual therapy combined with blood purification. The modalities employed included continuous veno-venous hemodiafiltration (CVVHDF) plus hemoperfusion (HP) (24, 23.3%); CVVHDF, HP, and plasma exchange (PE) (29, 28.1%); HP alone (7, 6.8%); CVVHDF alone (1, 1.0%); and HP plus PE (1, 1.0%) (Table 3).

**TABLE 3:** Clinical treatment and outcomes of patients with *Russula subnigricans* poisoning.

Characteristics	n	%
Ward		
General ward	93	90.3
Intensive care unit	6	5.8
Nephrology department	4	3.9
Treatment plan		
Usual care	41	39.8
Usual care and blood purification	62	60.2
Blood purification modality		
CVVHDF	1	1.0
CVVHDF and HP	24	23.3
CVVHDF, HP and PE	29	28.1
HP and PE	1	1.0
HP	7	6.8
Outcome		
Recovery	91	88.3
Death	12	11.7

CVVHDF, continuous veno-venous hemodiafiltration; HP, hemoperfusion; PE, plasma exchange.

Treatment regimens were titrated to individual clinical status, and medications were discontinued upon confirmation of clinical improvement and normalization of relevant laboratory parameters. No major adverse drug events were documented during the treatment course. After completion of care at Yunnan University Affiliated Hospital, the final clinical outcomes were as follows: 91 patients (88.3%) recovered and were discharged, while 12 patients (11.7%) died during hospitalization (Table 3).

Among the cohort, blood purification served as a key treatment modality, with 62 patients (60.2%) receiving at least one form of such therapy. Of these, 10 patients (16.1%) died despite blood purification (Table 4). A comparison of peak and pre-discharge laboratory values in these 62 patients showed improvement across all parameters, although liver function markers - AST and ALT - remained elevated above normal ranges (Table 5). This suggests that patients selected for blood purification based on the PSS criteria should be prioritized for this intervention to improve survival.

**TABLE 4:** Outcomes of patients undergoing blood purification therapy (n = 62).

Outcome	n	%
Survival	52	83.9
Death	10	16.1

**TABLE 5:** Comparison of peak and pre-discharge laboratory values in patients receiving blood purification (n = 62).

Para-meter	M (IQR:P25, P75) (n = 62)		Z	P	Normal range
	Peak value	Pre-discharge			
CK (U/L)	6718.50 (3570.00, 22000.00)	177.50 (72.00, 428.75)	−6.736^a^	<0.001	50.0–310.0
CK-MB (u/L)	500.00 (215.75, 1017.00)	24.00 (16.00, 52.25)	−6.567^a^	<0.001	0–25.0
MYO (ng/mL)	1200.00 (846.17, 1200.00)	75.35 (33.900, 194.82)	−6.624^a^	<0.001	0–140.1
Cr (umol/L)	73.50 (58.50, 104.50)	46.00 (56.000, 75.75)	−6.394^a^	<0.001	57.0–97.0
ALT (U/L)	318.50 (118.25, 703.00)	85.50 (42.750, 181.25)	−6.393^a^	<0.001	9.0–50.0
AST (U/L)	403.50 (144.50, 703.00)	43.00 (27.750, 77.00)	−6.451^a^	<0.001	15.0–40.0

Data are presented as median (interquartile range). CK (U/L): creatine kinase; CK-MB (u/L): creatine kinase; MYO (ng/mL): myoglobin; Cr (umol/L): creatinine; ALT (U/L): alanine aminotransferase; AST (U/L): aspartate aminotransferase.

^a^ Based on positive rank.

## 4 Discussion

Leveraging the rich mycological resources of Yunnan Province, we collected and analyzed medical records from 103 cases of highly suspected *Russula subnigricans* poisoning. This study represents the first systematic analysis of this relatively rare toxicological entity. Our findings indicate that the onset of *R. subnigricans* poisoning is typically early (<6 h), with only a small subset exhibiting delayed onset (>24 h). Initial clinical presentation most commonly involves gastrointestinal disturbances, including nausea and vomiting (85.4%), abdominal pain (18.4%), and diarrhea (20.4%). This is often followed by features of rhabdomyolysis, such as diffuse myalgia (52.4%) and fatigue. In severe cases, life-threatening complications emerge, including respiratory failure, acute kidney injury, electrolyte imbalances, hepatotoxicity, arrhythmias, myocardial injury, and shock, which may culminate in multi-organ failure and death.

Consequently, blood purification therapy is recommended for patients with moderate-to-severe poisoning. The mortality rate among highly suspected *R. subnigricans* poisoning patients admitted to our institution was only 11.7%, reflecting a relatively favorable therapeutic outcome. In emergency and critical care settings, early recognition and systematic supportive treatment are key determinants of prognosis. In this study, we observed that, despite the lack of a specific antidote, comprehensive intervention, including gastrointestinal decontamination, fluid resuscitation, organ support, and blood purification, significantly mitigated clinical deterioration. Lee et al. similarly emphasized that in regions with frequent mushroom outbreaks, mushroom poisoning should be considered in the differential diagnosis of rhabdomyolysis (Lee et al., 2001). Japanese case reports have noted that early recognition and aggressive supportive care may be crucial for survival in patients with *R. subnigricans* poisoning and rhabdomyolysis (Lin et al., 2015; Chun et al., 2023). A Korean case report described a fatal presentation of *R. subnigricans* poisoning characterized by rhabdomyolysis, acute kidney injury, severe hypocalcemia, respiratory failure, ventricular tachycardia, and cardiogenic shock, ultimately resulting in death (Cho and Han, 2016). Collectively, these findings suggest that early initiation of symptomatic therapy combined with blood purification can effectively salvage patients with moderate-to-severe poisoning, thus resulting in a favorable prognosis.

Laboratory testing revealed hepatic dysfunction as the most prevalent abnormality (97.1%), followed by elevated cardiac enzymes (85.4%), indicating substantial hepatotoxicity and myocardial involvement. In contrast, renal dysfunction was less common (23.3%), a finding that may be attributed to early medical intervention potentially preventing the progression of secondary kidney injury. Moreover, 65.1% of patients exhibited electrocardiographic abnormalities, most frequently ST-segment depression (39.8%) and ST-segment elevation (14.6%). Intermittent ventricular conduction blocks and tachycardia were also observed. These findings accord with postulated toxin mechanisms involving direct myocardial electrophysiological interference and ischemic injury, aligning with clinical features reported in Japanese and Korean case series.

Nevertheless, basic research and reliable identification methods for *R. subnigricans* remain limited. In 1992, Takahashi, a Japanese scholar, reported that *R. subnigricans* contains Russuphelin A, B, C, D, E, and F, compounds exhibiting cytotoxic activity against P388 leukemia cells (Takahashi et al., 1993). In 2009, Matsuura, a Japanese scholar, identified cycloprop-2-ene-carboxylic acid in this species as a causative agent of human rhabdomyolysis (Matsuura et al., 2009). Further work by Matsuura in 2016 led to the discovery of cyclopropanoyl-(R)-carnitine, a compound proposed for species-specific identification (Matsuura et al., 2016). In 2022, Long, a Chinese scholar, demonstrated the feasibility of identifying *R. subnigricans* using loop-mediated isothermal amplification (LAMP) of DNA (Long et al., 2022). Despite these advances, no clinically reliable laboratory test for *R. subnigricans* poisoning is currently available. Diagnosis still rests on anamnesis of mushroom ingestion, morphological assessment, and photographic recognition.

*Russula subnigricans* poisoning is now an established seasonal public-health threat in Yunnan. This study analyzed the epidemiological features, clinical management, and outcomes of this poisoning, providing evidence to inform public health prevention strategies and clinical decision-making. However, the precise etiology, toxin kinetics, toxic mechanisms, and dose-response relationship of *R. subnigricans* poisoning remain poorly elucidated. Future research should prioritize clarifying the toxi structure, pathophysiological pathways, and pharmacokinetic profile to enable the development of targeted antidotes and precision therapeutics.

### 4.1 Limitations

Additionally, this study excluded patients with significant pre-existing organ dysfunction or major chronic diseases to reduce confounding. While this approach enhances internal validity, it may limit external validity, as such comorbidities are frequently encountered in real-world emergency toxicology practice. The exact number of patients excluded based on these criteria was not systematically recorded; nonetheless, readers should consider that the findings may not be directly generalizable to poisoned patients with significant underlying comorbidities.

## Data Availability

The data used and analyzed during the current study are available from the corresponding author upon reasonable request. E-mail: m13888588958@163.com.
